# Repeatability and Reproducibility of Microscopic Examination of Adhesive Tape Strip Cytology Slides for the Diagnosis of *Malassezia* Overgrowth and/or *Malassezia* Dermatitis in Dogs

**DOI:** 10.3390/vetsci13070716

**Published:** 2026-07-21

**Authors:** Stathis Mpairamoglou, Dimitrios Tapes, Vassilis Skampardonis, Manolis K. Chatzis, Kosmas Apostolidis, Labrini V. Athanasiou, Dimitris Kasabalis, Kassiopi Christina G. Kokkinaki, Eleni G. Katsogiannou, Constantina N. Tsokana, Theodoros Petanides, Leonidas Leontides, Manolis N. Saridomichelakis

**Affiliations:** 1 Clinic of Medicine, Faculty of Veterinary Medicine, University of Thessaly, GR-43132 Karditsa, Greece; st.mpairamoglou@gmail.com (S.M.); jamestapes@yahoo.com (D.T.); mchatzis@uth.gr (M.K.C.); kapostol@uth.gr (K.A.); lathan@vet.uth.gr (L.V.A.); dikasvet@gmail.com (D.K.); kassikokkinaki@yahoo.gr (K.C.G.K.); elkatsog@uth.gr (E.G.K.); ctsokana@vet.auth.gr (C.N.T.); tpetanid@uth.gr (T.P.); 2 OmniVET Veterinary Clinic, GR-15125 Marousi, Greece; 3 Veterinary Clinic, Army Veterinary Training and Health Center, Stratigou N. Plastira, GR-41334 Larisa, Greece; 4 Laboratory of Epidemiology, Biostatistics and Animal Health Economics, Faculty of Veterinary Medicine, University of Thessaly, GR-43132 Karditsa, Greece; bskamp@uth.gr (V.S.); leoleont@uth.gr (L.L.); 5 Laboratory of Parasitology and Parasitic Diseases, School of Veterinary Medicine, Faculty of Health Sciences, Aristotle University of Thessaloniki, GR-54638 Thessaloniki, Greece

**Keywords:** cytology, dermatitis, *Malassezia*, overgrowth, repeatability, reproducibility

## Abstract

Adhesive tape strip cytology at 1000× magnification is the recommended method for the presumptive diagnosis of canine *Malassezia* overgrowth and/or dermatitis. This study aimed to determine the minimum number of microscopic fields required to achieve optimal intraobserver repeatability and interobserver reproducibility. Two groups of cytology slides were evaluated: 12 slides paired with photographs of pruritic dogs, in which a presumptive diagnosis of *Malassezia* dermatitis was defined by the detection of at least one yeast organism, and eight slides paired with photographs of non-pruritic dogs, in which a presumptive diagnosis of *Malassezia* overgrowth and dermatitis was defined by the detection of an average of at least one yeast organism per microscopic field. Twelve investigators examined each slide twice, and results from the first 10, 20, 30, 40, and 50 oil immersion fields were recorded and used for statistical analysis. Regardless of the number of microscopic fields examined, overall intraobserver repeatability and interobserver reproducibility were clinically non-acceptable for both slide groups and did not substantially increase in parallel with the number of the fields examined. Consequently, negative adhesive tape strip cytology results may not exclude *Malassezia* overgrowth and/or dermatitis in dogs with compatible clinical signs.

## Simple Summary

Yeasts of the genus *Malassezia* normally live on the skin of dogs, but in some cases, they can overgrow or trigger allergic reactions that lead to skin inflammation (dermatitis). In veterinary practice, cytology is commonly used to make a presumptive diagnosis of *Malassezia* dermatitis. This study investigated how many microscopic fields need to be examined to obtain consistent results when the same person evaluates the same slide twice, and when different people evaluate the same slide. Twelve investigators each examined 20 cytology slides twice. In 12 slides, a positive result was defined as detecting at least one yeast organism in total, while in eight slides it was defined as detecting an average of at least one yeast organism per microscopic field. Results from the first 10, 20, 30, 40, and 50 microscopic fields were compared. Regardless of the number of fields examined, agreement within and between investigators remained clinically non-acceptable for both groups of slides and did not substantially increase in parallel with the number of the fields examined. These findings suggest that negative cytology results do not rule out *Malassezia* dermatitis in dogs with compatible clinical signs.

## 1. Introduction

Yeasts of the genus *Malassezia*, particularly *M. pachydermatis*, are part of the normal cutaneous microbiota of dogs [1]. Under conditions that disrupt cutaneous homeostasis, such as integumentary or systemic disease, these organisms may proliferate excessively (*Malassezia* overgrowth), leading to pruritus and inflammation (*Malassezia* dermatitis) [1,2]. In dogs with atopic dermatitis (AD), an additional mechanism has been described whereby humoral and/or cellular hypersensitivity to *Malassezia* spp. allergens exacerbates pruritus and skin lesions, even in the absence of yeast overgrowth [1,2,3,4,5,6,7]. Consequently, current guidelines recommend that detection of any number of *Malassezia* organisms on the skin of dogs with AD should prompt a therapeutic trial to evaluate their potential role as flare factors [8].

In clinical practice, the presumptive diagnosis of *Malassezia* overgrowth and/or dermatitis relies primarily on cytological examination, with adhesive tape strip cytology being the most commonly used sampling technique [1,2,9,10]. We previously demonstrated that diagnostic accuracy improves when slides are examined at 1000× magnification and that the repeatability and reproducibility of yeast counts are poor, even after evaluation of 50 oil immersion fields (OIFs) [11]. However, the optimal number of OIFs required for reliable presumptive diagnosis of *Malassezia* overgrowth and/or dermatitis has not yet been established.

The aim of the present study was to determine the minimum number of OIFs that should be examined on adhesive tape strip cytology slides to achieve optimal intraobserver repeatability and interobserver reproducibility for the presumptive diagnosis of *Malassezia* overgrowth and/or dermatitis.

## 2. Materials and Methods

A total of 12 examiners participated in the study. Examiner 1 was a Diplomate of the European College of Veterinary Dermatology. Examiners 2–5 included a dermatology intern, clinic staff members, and PhD students with extensive experience in cutaneous cytology. Examiners 6–12 were less experienced and included three interns and four fourth- or fifth-year veterinary students. All examiners had participated in a previous study [11]; however, data from examiners 6–12 were not published because their participation had been intended solely for training purposes.

One week before the study, all examiners attended a 60-min training session conducted by examiner 1. A PowerPoint presentation was used to review the study objectives and design, the criteria for selecting OIFs, and the morphological features used for the identification of *Malassezia* spp. Examiners were instructed to evaluate OIFs located in the central area of the slide and containing moderate to high numbers of keratinocytes arranged in monolayers. The session concluded with a live demonstration of representative cytological preparations to standardize field selection and yeast identification using a microscope (BX-40 Olympus, Tokyo, Japan) equipped with an Altra 20 screen-projecting color camera (Olympus, Tokyo, Japan).

Twenty archived adhesive tape strip cytology slides obtained during routine clinical work-up were randomly selected from the archive of one of the authors and used in this study. Approximately 5-cm strips of transparent adhesive tape (Scotch Crystal Clear Tape, 3M, St. Paul, MN, USA) had been pressed 5–10 times onto lesional skin from dogs suspected of having *Malassezia* overgrowth and/or dermatitis. Tape strips were stained with Diff-Quik (Merck, Darmstadt, Germany) using the fixative and both staining solutions, air-dried, and mounted adhesive side down onto clean glass slides. Before the study, examiner 1 confirmed acceptable staining quality for all preparations. Slide identity was then masked, and slides were randomly numbered from 1 to 20 using an online random number generator (https://www.gigacalculator.com/calculators/random-number-generator.php: accessed on 26 February 2014).

Twenty high-quality clinical photographs showing lesions compatible with *Malassezia* dermatitis [1] were incorporated into a PowerPoint presentation. Each slide included one photograph and the text “Pruritus:” followed by either “yes” (12/20; 60%) or “no” (8/20; 40%). The primary diseases included AD (n = 5); demodicosis and flea allergic dermatitis (n = 2 each); and acral lick dermatitis, alopecia X, cyclic flank alopecia, dermatophytosis, digital trauma, follicular dysplasia, hyperadrenocorticism, hypothyroidism, leishmaniosis, muzzle folliculitis–furunculosis, and pemphigus foliaceus (n = 1 each). Photographs were cropped to minimize recognition of the primary disease.

The order of the photographs was randomized, and each image was assigned a number from 1 to 20 corresponding to a cytology slide bearing the same number. Therefore, cytology slides and photographs did not originate from the same dogs, preventing examiners from inferring the likely presence or absence of *Malassezia* organisms based on the clinical image.

Each examiner first viewed the clinical photograph and then examined the corresponding cytology slide at 1000× magnification. The numbers of *Malassezia* yeasts observed within the first 10, 20, 30, 40, and 50 OIFs were recorded. Examiners then determined whether a presumptive diagnosis of *Malassezia* overgrowth and/or dermatitis was justified after examination of each number of OIFs according to the following criteria: (a) detection of at least one yeast organism in skin pruritic lesions or (b) detection of an average of at least one yeast organism per OIF in non-pruritic skin lesions [12,13].

A second evaluation round was performed at least two weeks after completion of the first round. All cytological examinations (both first and second examination rounds) were performed using the same microscope (Carl Zeiss ICS-KF2 Binocular Research Microscope, Jena, Germany).

The number of cytology preparations included in the study was determined considering both the expected prevalence of *Malassezia* overgrowth and the feasibility of repeated examination of each slide by multiple observers. Considering that up to 74% of dogs with AD are sensitized to *Malassezia* allergens and therefore are likely to have *Malassezia* dermatitis (with or without *Malassezia* overgrowth) [14], even though the reported prevalence of cytologically detectable *Malassezia* overgrowth in dogs with AD is 38% [15], inclusion of 12 cytology preparations from pruritic dogs provides 80% power at a significance level of <0.05 to detect the 38% positive proportion (sample size calculation performed using the freely available online sample size calculator: https://clincalc.com/stats/samplesize.aspx: accessed on 14 February 2014). The prevalence of *Malassezia* overgrowth among non-allergic dogs is unknown. Therefore, considering the substantial time and effort required for repeated examination of each slide by 12 observers, inclusion of eight cytology preparations from non-allergic dogs was considered a reasonable and feasible compromise.

Statistical analyses were performed separately for slides paired with photographs of pruritic and non-pruritic dogs. Cohen’s kappa coefficient (*κ*) was calculated for the binary outcome variable (presumptive diagnosis of *Malassezia* overgrowth and/or dermatitis or not): (a) between the two examination rounds of the same examiner for the same slide and number of OIFs (intraobserver repeatability) and (b) between all examiner pairs evaluating the same slide, examination round, and number of OIFs (interobserver reproducibility). Overall interobserver reproducibility was assessed using Fleiss’ *κ*. Agreement was interpreted as poor (*κ* ≤ 0.20), fair (*κ*: 0.21–0.40), moderate (*κ*: 0.41–0.60), substantial (*κ*: 0.61–0.80), or good (*κ* > 0.80) [16]. For each observer (intraobserver repeatability) and each observer pair (interobserver reproducibility), percentage (%) agreement values obtained after examination of 10, 20, 30, 40, and 50 OIFs were compared using either one-way repeated measures ANOVA with Greenhouse–Geisser correction (normally distributed data) or Friedman repeated-measures ANOVA (non-normally distributed data) to evaluate changes in agreement across increasing numbers of OIFs. When significant differences were detected, post-hoc Wilcoxon signed-rank tests with Bonferroni correction were performed. Statistical analyses were conducted using SPSS 29 for Windows, and significance was set at *p* < 0.05.

Because only archived adhesive tape strip cytology slides obtained non-invasively during routine diagnostic procedures were used and no additional animal handling or sampling was involved, ethical approval was not required.

## 3. Results

Among the 12 cytology slides paired with photographs of pruritic dogs, the overall prevalence of a presumptive diagnosis of *Malassezia* dermatitis ranged from 49.7% after examination of 10 OIFs to 68.8% after examination of 50 OIFs (Table 1). Among the eight slides paired with photographs of non-pruritic dogs, the prevalence of a presumptive diagnosis of *Malassezia* overgrowth and dermatitis ranged from 9.9% to 12% depending on the number of OIFs examined (Table 2).

### 3.1. Intraobserver Repeatability

For slides paired with photographs of pruritic dogs, *κ* values ranged from −0.143 to 1 (Table 1). Agreement was classified as poor in 8/60 (13.3%), fair in 16/60 (26.7%), moderate in 5/60 (8.3%), substantial in 16/60 (26.7%), and good in 15/60 (25%) examiner/OIF number combinations (Table 3). Good repeatability was not achieved by 5/12 (41.7%) examiners at 10, 20, 30, 40, and 50 OIFs. The % agreement for each examiner after examination of 10, 20, 30, 40, and 50 OIFs is presented in Table 4. One-way repeated measures ANOVA with Greenhouse–Geisser correction showed no significant differences among examinations of 10, 20, 30, 40, and 50 OIFs (*p* = 0.516).

For slides paired with photographs of non-pruritic dogs, *κ* values ranged from −0.20 to 1 (Table 2). Agreement was classified as poor in 31/60 (51.7%), fair in 1/60 (1.7%), moderate in 3/60 (5%), substantial in none, and good in 25/60 (41.7%) examiner/OIF number combinations (Table 3). Good repeatability was not achieved by 4/12 (33.3%) examiners at 10, 20, 30, 40, and 50 OIFs. The % agreement for each examiner after examination of 10, 20, 30, 40, and 50 OIFs is presented in Table 5. Friedman repeated-measures ANOVA demonstrated no significant differences among examinations of 10, 20, 30, 40, and 50 OIFs (*p* = 0.227).

### 3.2. Interobserver Reproducibility

For slides paired with photographs of pruritic dogs, *κ* values ranged from −0.9 to 1. Agreement was classified as poor in 192/660 (29.1%), fair in 168/660 (25.5%), moderate in 145/660 (22%), substantial in 114/660 (17.3%), and good in 41/660 (6.2%) examiner pair/evaluation round/OIF number combinations (Table 6). Overall interobserver reproducibility was fair (Fleiss’ *κ* = 0.379; 95% confidence interval: 0.309–0.447).

Percentage agreement for each examiner pair/evaluation round ranged from 25% to 100%. Friedman repeated-measures ANOVA demonstrated no significant effect of the number of examined OIFs on % agreement (*p* = 0.163).

For slides paired with photographs of non-pruritic dogs, *κ* values ranged from −0.20 to 1. Agreement was classified as poor in 300/660 (45.5%), fair in 4/660 (0.6%), moderate in 73/660 (11.1%), substantial in none, and good in 283/660 (42.9%) examiner pair/evaluation round/OIF number combinations (Table 6). Overall interobserver reproducibility was moderate (Fleiss’ *κ* = 0.509; 95% confidence interval: 0.358–0.613).

Percentage agreement for each examiner pair/evaluation round ranged from 62.5% to 100%. Friedman repeated-measures ANOVA again identified a significant effect of the number of examined OIFs on *κ* values (*p* < 0.001). Post-hoc Wilcoxon signed-rank tests demonstrated significantly lower % agreement after examination of 10 OIFs compared with 40 (*p* < 0.01) and 50 OIFs (*p* < 0.01), and after examination of 20 OIFs compared with 10 (*p* = 0.02), 30 (*p* < 0.01), 40 (*p* < 0.01), and 50 OIFs (*p* < 0.01; *p* values were adjusted by the Bonferroni correction).

## 4. Discussion

For a cytological method to be clinically useful, it should provide results that are not only clinically meaningful, but also repeatable when assessed by the same observer and reproducible when assessed by different observers [16,17]. Despite the previously reported poor repeatability and reproducibility of adhesive tape strip cytology for quantitative yeast counts [11], we hypothesized that clinically acceptable agreement might still be achieved when cytology was used to diagnose *Malassezia* overgrowth and/or dermatitis. However, the present study demonstrated clinically non-acceptable overall intraobserver repeatability (Table 1, Table 2 and Table 3) and interobserver reproducibility (Table 6) for both diagnostic criteria evaluated: detection of at least one yeast organism in slides paired with photographs of pruritic dogs, and detection of an average of at least one yeast organism per OIF in slides paired with photographs of non-pruritic dogs.

A small number of examiners achieved consistently high repeatability regardless of the number of OIFs examined, particularly examiner #5 for pruritic cases (Table 1) and examiners #1, #2, and #5 for non-pruritic cases (Table 2). Although factors such as experience, visual acuity, and motivation may influence observer performance, the most likely explanation for the unsatisfactory overall agreement is the heterogeneous distribution of yeasts within the adhesive tape preparations, combined with the fact that different microscopic fields are examined during each evaluation round [18]. Increasing the number of examined OIFs could theoretically improve agreement; however, examination of 50 OIFs already requires approximately 4 min per slide [11], rendering further increases impractical for routine clinical use.

The prevalence of positive and negative tape strip cytology slides was relatively balanced among slides paired with clinical photographs of pruritic dogs (Table 1), whereas only approximately 10% of the slides paired with clinical photographs of non-pruritic dogs were classified as positive (Table 2). Although these proportions reflect clinical practice, where most cases of *Malassezia* overgrowth and/or dermatitis occur in pruritic dogs [1], the low prevalence of positive slides in the non-pruritic group may have artificially reduced Cohen’s κ coefficient, a phenomenon known as the prevalence paradox of kappa [19]. Because Cohen’s κ may underestimate the true level of agreement when category prevalence is markedly imbalanced, percentage agreement was also calculated. In the non-pruritic group in particular, percentage agreement was higher than the corresponding κ values, suggesting that κ underestimated the level of agreement between raters (Table 2 and Table 5).

Interestingly, in slides paired with photographs of non-pruritic dogs, interobserver % agreement improved when larger numbers of OIFs (30–50) were evaluated. A similar, although non-significant, trend was observed for intraobserver repeatability (Table 3). This likely reflects the diagnostic cut-off applied to this groups. For slides paired with photographs of non-pruritic dogs, positivity required an average of at least one yeast organism per OIF. In these cases, borderline yeast densities may have produced variable classifications after examination of relatively few fields, whereas evaluation of larger numbers of OIFs reduced variability around the diagnostic threshold.

This finding highlights the major influence of the selected diagnostic cut-off on both repeatability and reproducibility in the cytological diagnosis of canine *Malassezia* overgrowth and/or dermatitis. Differentiating between normal colonization and pathological overgrowth remains challenging, and a wide range of cut-offs has been proposed, including >1 to >5 yeasts per 400× high-power field and >0.7 to >4 yeasts per OIF [2,7,9,20,21,22]. These thresholds are largely derived from studies of healthy dogs; however, this approach has important limitations because normal yeast populations vary according to body site and breed [9,23,24]. Furthermore, detection of even small numbers of yeasts on clinically affected skin may be diagnostically relevant [25] particularly in dogs with AD that develop hypersensitivity to *Malassezia* spp. [1,8]. It is therefore possible that no universally applicable cytological cut-off exists for all body sites and underlying diseases, and that definitive diagnosis of *Malassezia* dermatitis ultimately depends on clinical response to antifungal therapy [2,26].

Previous studies have shown that approximately 50% of dogs with AD and a presumptive diagnosis of *Malassezia* dermatitis (diagnosed by the detection of at least one yeast organism on adhesive tape-strip cytology) respond to antifungal therapy, although, in that study, treatment was combined with antibacterial therapy in dogs with concurrent bacterial overgrowth or bacterial dermatitis [27]. Further studies are warranted to determine whether antifungal treatment can reduce AD severity in dogs in which no yeast organisms are detected after examination of 50 OIFs.

The present study has several limitations. First, the absence of a gold standard prevented assessment of the sensitivity and specificity of cytological examination and precluded determination of whether the observed disagreement resulted from observer error or from true uncertainty associated with the heterogeneous distribution of yeasts and the evaluation of different microscopic fields. Consequently, the underlying cause of the suboptimal repeatability and reproducibility observed in this study could not be established, limiting the clinical interpretation of our findings. Second, evaluation was limited to 50 OIFs; although examination of additional fields might have improved agreement, this would substantially reduce the practicality of the technique in routine clinical settings. Third, the findings of this study apply specifically to adhesive tape strip cytology. While different sampling methods, such as impression smears, skin scrapings, or swab smears, may show different levels of agreement, the objective of the present study was not to compare different sampling techniques but rather to assess the reliability of adhesive tape strip cytology, which is the most commonly used method for the diagnosis of *Malassezia* overgrowth and/or dermatitis in clinical practice. Fourth, this study was conducted at a single University Hospital using the same microscope, staining protocol, and examiner training. Therefore, the findings may not be directly generalized to other clinical settings, where differences in equipment, staining quality, examiner experience, and training may influence diagnostic performance and interobserver agreement.

## 5. Conclusions

Adhesive tape strip cytology showed clinically non-acceptable intraobserver repeatability and interobserver reproducibility for both the detection of a single yeast organism and the assessment of whether yeast density exceeded one organism per OIF, even after examination of 50 OIFs. Consequently, negative cytology results may not exclude *Malassezia* overgrowth and/or dermatitis in dogs with compatible clinical signs.

## Figures and Tables

**Table 1:** Cohen’s kappa coefficient (*κ*) and their 95% confidence intervals (in parentheses) for intraobserver repeatability in the presumptive diagnosis of *Malassezia* dermatitis following examination of 10, 20, 30, 40, and 50 oil immersion fields (OIFs) from 12 adhesive tape strip cytology slides paired with clinical photographs of pruritic dogs. Slides were evaluated twice at 1000× magnification by 12 examiners.

OIFs	Positive Slides (%) ^1^	Examiner											
		1	2	3	4	5	6	7	8	9	10	11	12
10	143/288 (49.7%)	0.625 (0.15–1)	0.5 (0.02–0.98)	**0.833** (0.53–1)	0.351 (−0.14–0.84)	**1** (1–1)	0.333 (−0.17–0.84)	0.385 (0–0.77)	0.385 (0–0.77)	0.333 (−0.17–0.84)	**1** (1–1)	0.063 (−0.41–0.54)	0.667 (0.25–1)
20	169/288 (58.7%)	**0.824** (0.5–1)	0.667 (0.27–1)	**1** (1–1)	0.333 (−0.17–0.84)	**0.833** (0.53–1)	0.351 (−0.14–0.84)	0.226 (0.17–0.62)	**0.833** (0.53–1)	0.063 (−0.41–0.54)	**0.824** (0.5–1)	0.25 (−0.37–0.87)	**0.833** (0.53–1)
30	183/288 (63.5%)	0.526 (0.12–0.94)	**0.824** (0.5–1)	0.667 (0.27–1)	0.636 (0.21–1)	**0.833** (0.53–1)	0.351 (−0.14–0.84)	0.308 (−0.18–0.8)	0.667 (0.27–1)	0.143 (−0.4–0.69)	**1** (1–1)	−0.125 (−0.3–0.05)	0.667 (0.27–1)
40	191/288 (66.3%)	0.676 (0.29–1)	0.636 (0.21–1)	0.5 (0.07–0.93)	0.636 (0.21–1)	**1** (1–1)	0.314 (−0.24–0.86)	0.308 (−0.18–0.8)	0.667 (0.27–1)	0.143 (−0.4–0.69)	**1** (1–1)	0 (0–0)	0.471 (−0.03–0.98)
50	198/288 (68.8%)	0.676 (0.29–1)	0.636 (0.21–1)	0.333 (−0.06–0.73)	0.636 (0.21–1)	**1** (1–1)	0.4 (−0.15–0.95)	0.308 (−0.18–0.8)	0.667 (0.27–1)	−0.143 (−0.37–0.08)	0.8 (0.43–1)	0 (0–0)	0.471 (−0.03–0.98)

Values shown in bold indicate good intraobserver repeatability (*κ* > 0.8). ^1^ Calculated as the number of the 12 cytology slides examined twice by each of the 12 examiners (12 × 2 × 12 = 288 examinations) in which at least one yeast organism was detected after examination of the first 10, 20, 30, 40, and 50 OIFs.

**Table 2:** Cohen’s kappa coefficient (*κ*) and their 95% confidence intervals (in parentheses) for intraobserver repeatability in the presumptive diagnosis of *Malassezia* overgrowth and dermatitis following examination of 10, 20, 30, 40, and 50 oil immersion fields (OIFs) from eight adhesive tape strip cytology slides paired with clinical photographs of non-pruritic dogs. Slides were evaluated twice at 1000× magnification by 12 examiners.

OIFs	Positive Slides (%) ^1^	Examiner											
		1	2	3	4	5	6	7	8	9	10	11	12
10	19/192 (9.9%)	**1** (1–1)	**1** (1–1)	0 (0–0)	0 (0–0)	**1** (1–1)	**1** (1–1)	0 (0–0)	0 (0–0)	0 (0–0)	−0.143 (−0.34–0.05)	−0.2 (−0.49–−0.09)	0 (0–0)
20	21/192 (10.9%)	**1** (1–1)	**1** (1–1)	0 (0–0)	0 (0–0)	**1** (1–1)	**1** (1–1)	0 (0–0)	−0.2 (−0.49–0.09)	0 (0–0)	0.6 (−0.07–1)	−0.2 (−0.49–−0.09)	0 (0–0)
30	23/192 (12%)	**1** (1–1)	**1** (1–1)	0 (0–0)	0 (0–0)	**1** (1–1)	**1** (1–1)	0 (0–0)	0.333 (−0.41–1)	0 (0–0)	0.6 (−0.07–1)	0 (0–0)	**1** (1–1)
40	19/192 (9.9%)	**1** (1–1)	**1** (1–1)	**1** (1–1)	0 (0–0)	**1** (1–1)	0 (0–0)	0 (0–0)	**1** (1–1)	0 (0–0)	0.6 (−0.07–1)	0 (0–0)	0 (0–0)
50	20/192 (10.4%)	**1** (1–1)	**1** (1–1)	**1** (1–1)	0 (0–0)	**1** (1–1)	**1** (1–1)	**1** (1–1)	**1** (1–1)	0 (0–0)	0 (0–0)	0 (0–0)	0 (0–0)

Values shown in bold indicate good intraobserver repeatability (*κ* > 0.8). ^1^ Calculated as the number of the eight cytology slides examined twice by each of the 12 examiners (8 × 2 × 12 = 192 examinations) in which an average of at least one yeast organism per OIF was detected after examination of the first 10, 20, 30, 40, and 50 OIFs.

**Table 3:** Categories of agreement based on Cohen’s kappa coefficient (*κ*) for intraobserver repeatability in the presumptive diagnosis of *Malassezia* overgrowth and/or dermatitis following examination of 10, 20, 30, 40, and 50 oil immersion fields (OIFs) from 12 adhesive tape strip cytology slides paired with clinical photographs of pruritic dogs and eight slides paired with clinical photographs of non-pruritic dogs. Slides were evaluated twice at 1000× magnification by 12 examiners. Each row represents the categories of intraobserver repeatability for the 12 examiners.

OIFs	Poor	Fair	Moderate	Substantial	Good
Detection of at least one yeast organism (cytology slides paired with clinical photographs of pruritic dogs)					
10	1/12 (8.3%)	5/12 (41.7%)	1/12 (8.3%)	2/12 (16.7%)	3/12 (25%)
20	1/12 (8.3%)	4/12 (33.3%)	0	1/12 (8.3%)	6/12 (50%)
30	2/12 (16.7%)	2/12 (16.7%)	1/12 (8.3%)	4/12 (33.3%)	3/12 (25%)
40	2/12 (16.7%)	2/12 (16.7%)	2/12 (16.7%)	4/12 (33.3%)	2/12 (16.7%)
50	2/12 (16.7%)	3/12 (25%)	1/12 (8.3%)	5/12 (41.7%)	1/12 (8.3%)
Detection of an average of at least one yeast organism per OIF (cytology slides paired with clinical photographs of non-pruritic dogs)					
10	8/12 (66.7%)	0	0	0	4/12 (33.3%)
20	7/12 (58.3%)	0	1/12 (8.3%)	0	4/12 (33.3%)
30	5/12 (41.7%)	1/12 (8.3%)	1/12 (8.3%)	0	5/12 (41.7%)
40	6/12 (50%)	0	1/12 (8.3%)	0	5/12 (41.7%)
50	5/12 (41.7%)	0	0	0	7/12 (58.3%)

**Table 4:** Percentage agreement (%) for each of 12 examiners in the presumptive diagnosis of *Malassezia* dermatitis following examination of 10, 20, 30, 40, and 50 oil immersion fields (OIFs) from 12 adhesive tape strip cytology slides paired with clinical photographs of pruritic dogs. Slides were evaluated twice at 1000× magnification by each examiner.

OIFs	Examiner											
	1	2	3	4	5	6	7	8	9	10	11	12
10	83.3	75	91.7	66.7	100	66.7	66.7	66.7	66.7	100	58.3	83.3
20	91.7	83.3	100	66.7	91.7	66.7	66.7	91.7	58.3	91.7	75	91.7
30	75	91.7	83.3	83.3	91.7	66.7	75	83.3	66.7	100	75	83.3
40	83.3	83.3	75	83.3	100	66.7	75	83.3	66.7	100	83.3	75
50	83.3	83.3	66.7	83.3	100	75	75	83.3	66.7	91.7	83.3	75

**Table 5:** Percentage agreement (%) for each of the 12 examiners in the presumptive diagnosis of *Malassezia* overgrowth and dermatitis following examination of 10, 20, 30, 40, and 50 oil immersion fields (OIFs) from eight adhesive tape strip cytology slides paired with clinical photographs of non-pruritic dogs. Slides were evaluated twice at 1000× magnification by each examiner.

OIFs	Examiner											
	1	2	3	4	5	6	7	8	9	10	11	12
10	100	100	87.5	87.5	100	100	87.5	87.5	87.5	75	62.5	87.5
20	100	100	87.5	87.5	100	100	87.5	62.5	87.5	87.5	62.5	87.5
30	100	100	87.5	87.5	100	100	87.5	75	87.5	87.5	75	100
40	100	100	100	87.5	100	87.5	87.5	100	87.5	87.5	87.5	87.5
50	100	100	100	87.5	100	100	100	100	87.5	75	87.5	87.5

**Table 6:** Categories of agreement based on Cohen’s kappa coefficient (*κ*) for interobserver reproducibility in the presumptive diagnosis of *Malassezia* overgrowth and/or dermatitis following examination of 10, 20, 30, 40, and 50 oil immersion fields (OIFs) from 12 adhesive tape strip cytology slides paired with clinical photographs of pruritic dogs and eight slides paired with clinical photographs of non-pruritic dogs. Slides were evaluated twice at 1000× magnification by 12 examiners. Each row represents the categories of intraobserver reproducibility for the 132 pairs of examiners.

OIFs	Poor	Fair	Moderate	Substantial	Good
Detection of at least one yeast organism (cytology slides paired with clinical photographs of pruritic dogs)					
10	32/132 (24.2%)	35/132 (26.5%)	32/132 (24.2%)	18/132 (13.6%)	15/132 (11.4%)
20	37/132 (28%)	36/132 (27.3%)	21/132 (15.9%)	24/132 (18.2%)	14/132 (10.6%)
30	38/132 (28.8%)	35/132 (26.5%)	29/132 (22%)	25/132 (18.9%)	5/132 (3.8%)
40	40/132 (30.3%)	30/132 (22.7%)	31/132 (23.5%)	26/132 (19.7%)	5/132 (3.8%)
50	45/132 (34.1%)	32/132 (24.2%)	32/132 (24.2%)	21/132 (15.9%)	2/132 (1.5%)
Detection of an average of at least one yeast organism per OIF (cytology slides paired with clinical photographs of non-pruritic dogs)					
10	76/132 (57.6%)	0	7/132 (5.3%)	0	49/132 (37.1%)
20	71/132 (53.8%)	1/132 (0.8%)	16/132 (12.1%)	0	44/132 (33.3%)
30	45/132 (34.1%)	3/132 (2.3%)	31/132 (23.5%)	0	53/132 (40.2%)
40	55/132 (41.7%)	0	10/132 (7.6%)	0	67/132 (50.8%)
50	53/132 (40.2%)	0	9/132 (6.8%)	0	70/132 (53%)

## Data Availability

Raw data are available by the corresponding author after reasonable request.

## Abbreviations

The following abbreviations are used in this manuscript:

AD	Atopic dermatitis
ANOVA	Analysis of variance
OIF	Oil immersion field

## References

[1] Bond R., Morris D.O., Guillot J., Bensignor E.J., Robson D., Mason K.V., Kano R., Hill P.B. (2020). Biology, diagnosis and treatment of *Malassezia* dermatitis in dogs and cats. Clinical consensus guidelines of the World Association for Veterinary Dermatology. Vet. Dermatol..

[2] Chen T.A., Hill P.B. (2005). The biology of *Malassezia* organisms and their ability to induce immune responses and skin disease. Vet. Dermatol..

[3] Meason-Smith C., Diesel A., Patterson A.P., Older C.E., Mansell J.M., Suchodolski J.S., Rodrigues Hoffmann A. (2015). What is living on your dog’s skin? Characterization of the canine cutaneous mycobiota and fungal dysbiosis in canine allergic dermatitis. FEMS Microbiol. Ecol..

[4] Meason-Smith C., Olivry T., Lawhon S.D., Rodrigues Hoffmann A. (2020). *Malassezia* species dysbiosis in natural and allergen-induced atopic dermatitis in dogs. Med. Mycol..

[5] Plant J.D., Rosenkrantz W.S., Griffin C.E. (1992). Factors associated with and prevalence of high *Malassezia pachydermatis* numbers on dog skin. J. Am. Vet. Med. Assoc..

[6] Farver K., Morris D.O., Shofer F., Esch B. (2005). Humoral measurement of type-1 hypersensitivity reactions to a commercial *Malassezia* allergen. Vet. Dermatol..

[7] Nuttall T.J., Halliwell R.E. (2001). Serum antibodies to *Malassezia* yeasts in canine atopic dermatitis. Vet. Dermatol..

[8] Hensel P., Santoro D., Favrot C., Hill P., Griffin C. (2015). Canine atopic dermatitis: Detailed guidelines for diagnosis and allergen identification. BMC Vet. Res..

[9] Bensignor E., Jankowski F., Seewald W., Touati F., Deville M., Guillot J. (2002). Comparison of two sampling techniques to assess quantity and distribution of *Malassezia* yeasts on the skin of Basset Hounds. Vet. Dermatol..

[10] Omodo-Eluk A.J., Baker K.P., Fuller H. (2003). Comparison of two sampling techniques for the detection of *Malassezia pachydermatis* on the skin of dogs with chronic dermatitis. Vet. J..

[11] Tapes D., Skampardonis V., Chatzis M.K., Apostolidis K., Athanasiou L.V., Kasabalis D., Kokkinaki K.C.G., Katsogiannou E.G., Petanides T., Leontides L. (2022). Repeatability and reproducibility of microscopic examination of adhesive tape strip cytology slides for the quantification of *Malassezia* spp. in canine skin. Vet. Dermatol..

[12] Pinchbeck L.R., Hillier A., Kowalski J.J., Kwochka K.W. (2002). Comparison of pulse administration versus once daily administration of itraconazole for the treatment of *Malassezia pachydermatis* dermatitis and otitis in dogs. J. Am. Vet. Med. Assoc..

[13] Morris D.O., O’Shea K., Shofer F.S., Rankin S. (2005). *Malassezia pachydermatis* carriage in dog owners. Emerg. Infect. Dis..

[14] Morris D.O., Clayton D.J., Drobatz K.J., Felsburg P.J. (2002). Response to *Malassezia pachydermatis* by peripheral blood mononuclear cells from clinically normal and atopic dogs. Am. J. Vet. Res..

[15] Zur G., Ihrke P.J., White S.D., Kass P.H. (2002). Canine atopic dermatitis: A retrospective study of 266 cases examined at the University of California, Davies, 1992–1998. Part I. Clinical features and allergy testing results. Vet. Dermatol..

[16] Petrie A., Watson P. (2013). Statistics for Veterinary and Animal Science.

[17] McHugh M.L. (2012). Interrater reliability: The kappa statistic. Biochem. Medica.

[18] Budach S.C., Mueller R.S. (2012). Reproducibility of a semiquantitative method to assess cutaneous cytology. Vet. Dermatol..

[19] Feinstein A.R., Cicchetti D.V. (1990). High agreement but low kappa: I. The problems of two paradoxes. J. Clin. Epidemiol..

[20] Chen T.A., Halliwell R.E., Pemberton A.D., Hill P.B. (2002). Identification of major allergens of *Malassezia pachydermatis* in dogs with atopic dermatitis and *Malassezia* overgrowth. Vet. Dermatol..

[21] Rosales M.S., Marsella R., Kunkle G., Harris B.L., Nicklin C.F., Lopez J. (2005). Comparison of the clinical efficacy of oral terbinafine and ketoconazole combined with cephalexin in the treatment of *Malassezia* dermatitis in dogs-a pilot study. Vet. Dermatol..

[22] Berger D.J., Lewis T.P., Schick A.E., Stone R.T. (2012). Comparison of once-daily versus twice-weekly terbinafine administration for the treatment of canine *Malassezia* dermatitis—A pilot study. Vet. Dermatol..

[23] Bond R., Lloyd D.H. (1997). Skin and mucosal populations of *Malassezia pachydermatis* in healthy and seborrhoeic Basset Hounds. Vet. Dermatol..

[24] Bond R., Saijonmaa-Koulumies L.E., Lloyd D.H. (1995). Population sizes and frequency of *Malassezia pachydermatis* at skin and mucosal sites on healthy dogs. J. Small Anim. Pract..

[25] Patterson A.P., Frank L.A. (2002). How to diagnose and treat *Malassezia* dermatitis in dogs. Vet. Med..

[26] Mason K.V., Evans A.G. (1991). Dermatitis associated with *Malassezia pachydermatis* in 11 dogs. J. Am. Anim. Hosp. Assoc..

[27] Sofou E.I., Aleksandrova S., Badulescu E., Chatzis M., Saridomichelakis M. (2022). Efficacy of antimicrobial treatment in dogs with atopic dermatitis: An observational study. Vet. Sci..

